# Exploring the Dynamics of Charge Transfer in Photocatalysis: Applications of Femtosecond Transient Absorption Spectroscopy

**DOI:** 10.3390/molecules29173995

**Published:** 2024-08-23

**Authors:** Na Li, Yanlong Ma, Wanjun Sun

**Affiliations:** 1 School of New Energy and Power Engineering, Lanzhou Jiaotong University, Lanzhou 730070, China; 2 College of Chemistry and Chemical Engineering, Lanzhou University, Lanzhou 730000, China; 220220925031@lzu.edu.cn

**Keywords:** ultrafast spectroscopy, charge transfer mechanism, photocatalysis

## Abstract

Artificial photocatalytic energy conversion is a very interesting strategy to solve energy crises and environmental problems by directly collecting solar energy, but low photocatalytic conversion efficiency is a bottleneck that restricts the practical application of photocatalytic reactions. The key issue is that the photo-generated charge separation process spans a huge spatio-temporal scale from femtoseconds to seconds, and involves complex physical processes from microscopic atoms to macroscopic materials. Femtosecond transient absorption (fs-TA) spectroscopy is a powerful tool for studying electron transfer paths in photogenerated carrier dynamics of photocatalysts. By extracting the attenuation characteristics of the spectra, the quenching path and lifetimes of carriers can be simulated on femtosecond and picosecond time scales. This paper introduces the principle of transient absorption, typical dynamic processes and the application of femtosecond transient absorption spectroscopy in photocatalysis, and summarizes the bottlenecks faced by ultrafast spectroscopy in photocatalytic applications, as well as future research directions and solutions. This will provide inspiration for understanding the charge transfer mechanism of photocatalytic processes.

## 1. Introduction

Atmospheric warming, energy shortage and environmental pollution have become major scientific, technological and social problems restricting the sustainable development of human society [1,2,3,4]. Based on this, achieving the goal of “double carbon” is not only the urgent need of the current global social development, but also the key to deal with the challenge of climate change [2]. Photocatalytic reactions triggered by solar energy are one of the most potential energy conversion methods [5,6]. But its efficiency is low, which is mainly restricted by three aspects: light absorption, separation and migration of photogenerated carriers and surface catalytic reaction. The separation and migration of photogenerated carriers is the key to affect the efficiency of photocatalysis [7]. The basic problem is that the time scale from photoexcitation to surface chemical reaction spans 10 orders of magnitude, how to efficiently separate the photogenerated charge at the micro-nano scale and transfer it to the catalyst surface to drive the catalytic reaction [8]. So, in order to obtain more efficient photocatalysts, it is very important to understand the carrier dynamics process of photocatalysts after photoexcitation [9].

The common fast kinetic processes include carrier diffusion in semiconductor materials, carrier radiation recombination in luminescent materials, charge separation in catalytic reaction and so on [10]. The understanding of these dynamic processes is of great significance to the research and development of material properties. Ultrafast laser spectroscopy is a branch of spectroscopy that uses ultrafast laser to carry out experiments. As many dynamic processes in materials occur in a very short time range, tracking these processes requires ultra-high time resolution, but the response time of traditional electronic instruments is difficult to meet the requirements of ultrafast process research [11]. Professor Zewail used femtosecond spectroscopy to study the transition states of chemical reactions, which had a great impact on basic chemistry and physics, and led to the birth of a new discipline of femtosecond chemistry [12,13]. The appearance of femtosecond pulsed laser greatly improves the time resolution of ultrafast laser spectrum, which makes it possible to observe the ultrafast process at the atomic level after photon absorption, which provides a powerful weapon for the study of ultrafast dynamics. Compared with time-resolved fluorescence spectra, transient absorption spectra can be used to study the evolution of non-radiative processes and dark states. Femtosecond transient absorption spectra (fs-TA) can not only provide rich photophysical and photochemical process information for the study of one-component systems, but also be used to study the charge transfer and energy transfer processes between composites [14,15,16,17]. This ultrafast photonics related processes have important applications in the study of photocatalysis, solar cells (photovoltaic devices), LED, nonlinear optics, photodetectors and other fields, and can provide theoretical support and experimental verification for the selection of device materials and the reasonable design of device structures.

This article introduces the principle of transient absorption, typical kinetic processes and its application in photocatalytic processes. In short, it mainly includes a summary of the charge transfer kinetic processes of some common semiconductors, such as CdS, TiO_2_, g-C_3_N_4_, BiVO_4_, perovskites and MOF composites, in photocatalytic water decomposition and CO_2_ reduction. The current problems and future directions faced by ultrafast spectroscopy in photocatalytic applications are summarized. This review may provide a reference and guidance for future research to understand the principles and applications of femtosecond transient absorption and provide forward-looking guidance for the interpretation of charge transfer processes in photocatalysis.

## 2. Kinetics of Excited States of Photocatalysts

### 2.1. The Principle of Transient Absorption

**Figure 1:**
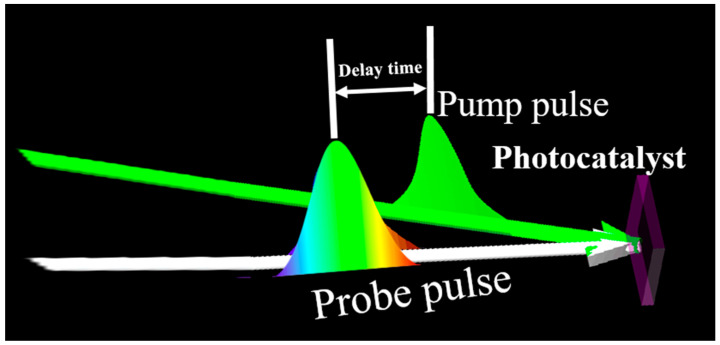
Schematic diagram of femtosecond transient absorption based on “pump-probe” technology.

The transient absorption spectra based on “pump-probe” technology is a powerful tool, which uses ultrafast laser pulses to study the photophysical properties of substances in a very short time [18,19]. In this method, both the pump beam and the probe beam originate from the same laser pulse, and the probe continuum pulse is delayed relative to the excitation pulse. The pump pulse is used to excite the sample. When the sample is excited, its physical or chemical properties will change and some transient intermediates may be generated. The probe pulse is used to detect the absorption of the excited sample. The transient absorption spectra can be obtained from the sample absorption difference [20] (Figure 1). By comparing the instantaneous changes of the sample signal, the reaction path and kinetic process of the sample can be obtained. Specifically, what appears in the transient absorption spectra is the change in the intensity of absorbed or reflected light in the sample and forms a signal on a specific time scale, that is, the photophysical behavior of the sample on an ultra-fast time scale. The pump light of transient absorption spectroscopy is mostly visible light, and the probe light includes visible light, near infrared light, medium infrared light and terahertz (THz) light according to different research processes and objects. Visible light is mainly used to study the changes of electrons between different excited states, such as the transition from valence band to conduction band and the dynamic process of excitons from ground state to excited state. Near-infrared light gives the dynamics of free carriers and the transition dynamics between energy levels of defect states, while mid-infrared light detects the interaction between free carriers and excitons, and terahertz light provides the dynamic process of free carriers and phonons [21]. The dynamic process of the material can also be obtained by selecting pump light in other bands according to the research object [22].

From the absorption formula, the absorption A_pump-on_ of the sample excited by pump light can be obtained as follows: A_pump-on_ = −lg(I_pump-on_/I_0_), the absorption A_pump-off_ of the sample which is not excited by pump light is: A_pump-off_ = −lg(I_pump-off/I0_). Therefore, the change of absorption ΔA_n_ is: ΔA = A_pump-on_ − A_pump-off_ = lg(I_pump-off_/I_pump-on_). In the formula, I_pump-on_ and I_pump-off_ are the detection light intensity with and without pump light excitation respectively, and I_0_ is the probe light intensity without sample. For the above, it can be inferred that with the change of time after the sample is excited by light, the transient absorption spectra of the sample at different detection wavelengths will also change. By changing the delay time between the probe light and the pump light, these changes can be observed, and the kinetic information of the molecules in the sample can be further obtained.

In the transient absorption spectra, several different signals are often observed [20]. Among them, the ground state bleaching (GSB) signal refers to the negative ΔA signal produced by the corresponding reduction of the ground state absorption due to the excitation of some molecules from the ground state to the excited state caused by pumping light. While stimulated radiation (SE) signal is a positive ΔA signal produced by a photon when the wavelength of the detected photon is the same as the excited state energy difference of the sample, and the excited sample returns to the ground state by stimulated radiation. In addition, excited state absorption (ESA) signal means that when the sample is in the excited state, it can still absorb the photons and form a positive ΔA signal. By fitting the signals, the kinetic information of different excited states can be obtained to understand the physical and chemical mechanism in the sample. Transient absorption spectroscopy is widely used to study rapid kinetic processes in chemistry, biology and material science, such as laser-induced kinetic processes, free radical reactions, charge transfer, polymer formation, electron transfer and so on [23].

### 2.2. Ultrafast Dynamic Process

When a molecule is excited to an excited state, a series of photochemical or photophysical processes can occur within and between molecules [24,25]. These processes include chemical bond breaking, bonding, intramolecular energy transfer, charge transfer, molecular vibration relaxation, intermolecular energy transfer, charge transfer, singlet relaxation, triplet relaxation and so on [26]. The properties and relaxation processes of these excited states as well as the optical absorption process can be described by Jablonski diagram [27]. Under light excitation, the molecule transitions from the ground state S_0_ to the first excited state S_1_ or higher S_2_-S_n_ by absorbing photons. The excited molecule returns to the first excited state S_1_ through internal conversion and vibrational relaxation, and then returns to the ground state by radiative transition (fluorescence, delayed fluorescence) and non-radiative transition (internal conversion, vibrational relaxation, intersystem crossing). The common dynamic processes are as follows:

Internal conversion is a non-radiative recombination process between two electron levels with the same spin multiplicity. After being excited by light, the molecule transitions from the ground state S_0_ to the excited state S_n_ whose energy is higher than the first electron excited state S_1_, the excited molecule can quickly undergo internal conversion relaxation to S_1_, and then reach the lowest vibrational energy level of S_1_ by vibrational relaxation, and finally return to the ground state S_0_. The internal conversion process is generally fast, and the time scale is about femtosecond to picosecond. Because the energy difference between S_1_ and S_0_ is much larger than that between S_n_ and S_1_, the internal conversion efficiency from S_1_ state to S_0_ state is lower than that between other excited states.

The photons emitted by the molecule directly from the excited singlet state S_1_ to the ground state S_0_ by radiation recombination are called fluorescence. The wavelength of fluorescence is generally determined by the energy difference between energy levels and is not affected by the wavelength of excited light. Due to the existence of vibrational energy levels in the molecule, there can be a stokes shift between the position of the fluorescence peak and the first absorption edge. The fluorescence intensity of the molecule attenuates exponentially with time, and the decay time can reflect the lifetime of the molecule in the excited state, generally in the order of picosecond to nanosecond. Intersystem crossing is a non-radiative transition process that occurs between electronic energy levels with different spin multiplicities. For example, a molecule in the singlet state S_1_ can transition to the triplet state T_1_. According to the selection rule, transitions between energy levels with different spin multiplicities are prohibited, while intersystem crossings can be achieved in molecules with strong spin-orbit coupling, and the time scale of this occurs is picoseconds to nanoseconds. The molecule in the triplet state T_1_ can return directly to the ground state S_0_ by radiation recombination and non-radiation recombination, in which the photons emitted by radiation recombination are called phosphorescence. Because the transition from T_1_ to S_0_ is blocked, the lifetime of phosphorescence is much longer than that of fluorescence, which can reach the order of milliseconds or even seconds. In addition to returning directly to the ground state S_0_, the molecule in the triplet state can also reach the singlet state S_1_ and then return to the ground state S_0_, in which case delayed fluorescence can be produced.

### 2.3. Kinetic Process of Photocatalyst

A variety of techniques have been used to study the carrier dynamics of photocatalysts. For example, the surface photovoltage microscope (SPVM) with high spatial and energy resolution recently reported by Li’s group, which can directly draw the surface charge distribution map and quantitatively evaluate the charge separation characteristics of photocatalysts on the nanometer scale, which may provide unprecedented insights into the photocatalytic charge separation process [28]. Some in situ techniques, such as in situ irradiation X-ray photoelectron spectroscopy and in situ electron paramagnetic resonance spectroscopy, can be used to reveal the physical and chemical behavior of photogenerated charges. Among these techniques, time-resolved infrared spectroscopy (TRIR) and transient absorption spectroscopy (TAS) have been widely used to obtain information such as charge transfer dynamics on the femtosecond to second time scale [21]. For photocatalytic reactions, the carrier dynamics during the photocatalytic process are characterized by transient absorption spectroscopy, and other in situ techniques are used to detect the conversion process of some specific intermediates, such as capturing intermediates in the photocatalytic CO_2_ conversion process through time-resolved infrared spectroscopy. In other words, TAS and TRIR can describe the kinetic process of carriers and the reaction path of reactants. After the charge transfer kinetics and intermediate steps are clarified, it provides a solid foundation for the rational design of photocatalysts [29,30].

For semiconductor materials, when excited by photons whose energy is greater than the band gap, the electrons in the valence band absorb photon energy and transition to the conduction band, while holes are generated in the valence band. The carriers produced by optical excitation relax to the corresponding band bottom through the interaction with other carriers and phonons, and finally return to the equilibrium state after different time and space evolution [18,20,31]. It often contains impurities or defects, so that the defect state energy level is introduced into the semiconductor bandgap. This defect state energy level can capture non-equilibrium carriers, which has an effect on the carrier relaxation dynamics. Different types of defect states have different effects on the carrier lifetime, and the recovery time ranges from a few picoseconds to hundreds of picoseconds, or even nanoseconds or milliseconds. However, the excited state in semiconductors does not always exist in the form of free carriers. In systems of quantum dots and two-dimensional materials, because of the quantum confinement effect, electrons and holes are bound together to form excitons because of coulomb interaction. Therefore, the exciton dynamics is commonly used to describe the system [32].

**Figure 2:**
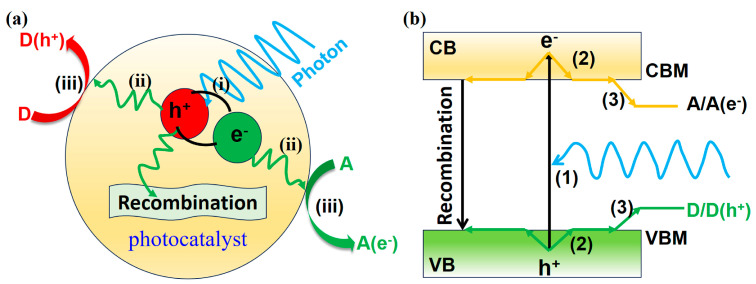
Under light excitation (**a**) inorganic semiconductors may have processes (**b**) transfer paths of electrons and holes in inorganic semiconductors.

Different types of semiconductors have different carrier transport processes (electron and hole transfer dynamics) [21]. For inorganic semiconductors, the light-driven charge transfer is almost linear (Figure 2a,b). After being excited by light, the following processes usually occur: (i) exciton generation, (ii) carrier separation and diffusion to the surface reaction site, and (iii) electron or hole reduction of electron acceptor (A) or oxidized donor (D). However, for organic semiconductors, it may involve the formation of long-lived triplet states. After light excitation, intersystem crossings, annihilation of triplet states, and corresponding recombination processes usually occur.

Therefore, TAS has been widely used to study carrier trapping, charge transfer between semiconductors and auxiliary catalysts, charge separation between heterojunctions, recombination kinetics and interfacial charge transfer between photocatalysts and chemical reactants.

## 3. Application of Transient Absorption Spectroscopy in Photocatalysis

### 3.1. Ultrafast Kinetics of Common Semiconductors in Photocatalysis

**Figure 3:**
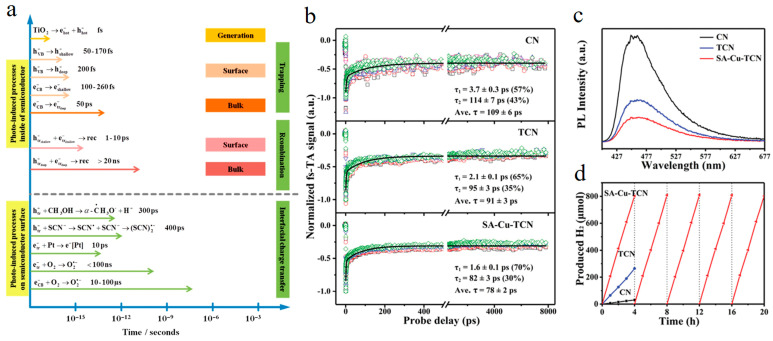
(**a**)The photolysis mechanisms of the following three typical molecules on the surface of TiO_2_ single crystal (mainly on R-TiO_2_) are studied: oxygen (O_2_), H_2_O and methanol (CH_3_OH) (Reproduced with permission [37], Copyright 2014, American Chemical Society). (**b**) Kinetic decay curves, (**c**) pL spectra and (**d**) hydrogen production performance for CN, TCN, and SA-Cu–TCN (Reproduced with permission [42], Copyright 2020, WILEY-VCH).

As a clean, environmentally friendly and pollution-free gas, hydrogen is considered to be a promising source of energy [33,34,35,36]. In 1972, the first metal oxide TiO_2_ which can be used to catalyze water splitting was reported, and a large number of literatures have given the carrier dynamics process of TiO_2_-based photocatalysts [37]. Unfortunately, about 90% of the photogenerated electron-hole pairs of the TiO_2_ catalyst recombine after absorbing light, and less than 10% of the electrons or holes are separated [38,39]. Therefore, improving the charge separation efficiency of photocatalysts has always been the focus of research, such as changing the morphology of TiO_2_ from nanoparticles to one-dimensional (1D) structure, and constructing heterojunctions, phase junctions and schottky junctions with other materials to solve the above problems [40]. Watanabe and Hayashi proposed, hot electrons and deep holes are more likely to separate than the charges generated by the band edge. In addition, the application of electric potential can also enhance the charge separation [41]. Schneider et al. summarized the time scale of carrier generation, heating, capture, recombination and transfer of TiO_2_ in photocatalysis [37] (Figure 3a). Focus on the photocatalysis mechanism of the following three typical molecules on the surface of TiO_2_ single crystals (oxygen (O_2_), H_2_O and methanol (CH_3_OH)). O_2_ usually plays a key role in the photocatalytic degradation of harmful organic pollutants on TiO_2_ catalyst, which is manifested in its high affinity for electrons and is often used to enhance electron-hole separation. The thermal and photochemical reactions of CH_3_OH on the TiO_2_ surface also have potential applications (including reforming to H_2_ and biomass conversion) and are also important for understanding the basic process of TiO_2_ catalysis.

Hematite (α-Fe_2_O_3_) has made great progress as a potentially practical and sustainable material in applications such as solar energy conversion and photoelectrochemical (PEC) water splitting [43,44,45,46]. Zhang et al. used femtosecond transient absorption (fs-TA) spectroscopy to study Fe_2_O_3_ nanoparticles with different shapes to determine the effect of particle shape on exciton dynamics [47]. Although the overall optical absorption and scattering are affected by the particle morphology, the overall exciton lifetime of Fe_2_O_3_ is basically the same. This study suggests that other strategies need to be developed to increase exciton lifetimes in Fe_2_O_3_ nanostructures or extend exciton diffusion lengths to improve performance.

In addition to the applications of TiO_2_ and Fe_2_O_3_ in the field of energy conversion, the non-metallic photocatalyst g-C_3_N_4_ has attracted the interest of many researchers due to its easy synthesis, abundant storage, physical and chemical stability, and response to visible light [48,49,50]. However, the light conversion efficiency is far lower than that of industrial applications due to its wide band gap, serious carrier recombination, and lack of surface active sites [51,52]. The most important thing is that the shortened electron relaxation time due to the decay of the lifetime of active species is detrimental to the improvement of photocatalytic activity [53].

Introducing atomic-scale metals is considered to be one of the effective ways to improve the charge separation and transport capabilities of g-C_3_N_4_ [54]. Fu et al. proposed that anchoring metal monatomic Cu into the C_3_N_4_ layer can effectively improve charge transport within the layer [42]. Ultrafast absorption spectroscopy further confirmed that Cu-N_x_ can significantly improve the transmission speed of photogenerated charges of C_3_N_4_ layers (Figure 3b). Therefore, the catalyst exhibits excellent visible light catalytic hydrogen evolution performance, which is 30 times that of bulk C_3_N_4_ (Figure 3c,d). This work has enabled to better understand the interaction between metal single atoms and C_3_N_4_ and its influence on the mechanical process of charge transport, thus providing new research ideas for the development of single-atom photocatalytic and photosynthesis.

**Figure 4:**
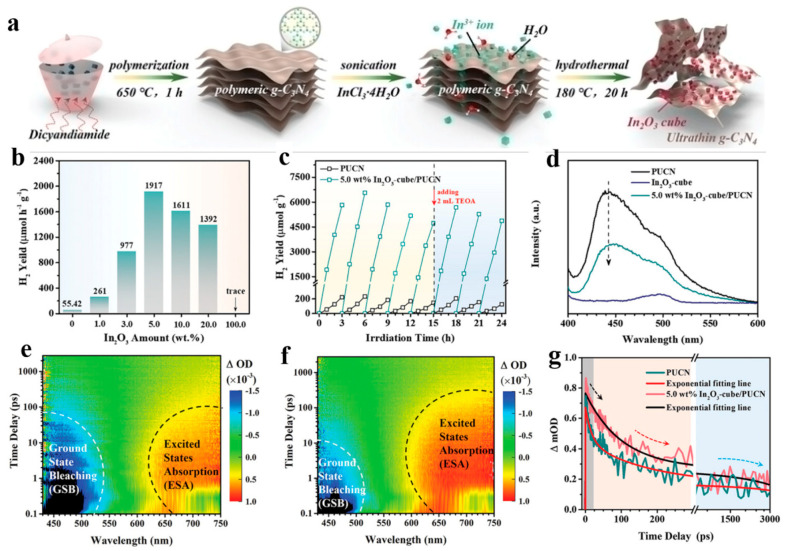
(**a**) Synthesis schematic of In_2_O_3_/PUCN composite, (**b**) Hydrogen production performance of different catalysts, (**c**) Photocatalytic stability of PUCN (black) and 5.0 wt% In_2_O_3_-cube/PUCN (cyan) catalysts (**d**) Fluorescence spectra of PUCN (black), In_2_O_3_-cube (blue) and 5.0 wt% In_2_O_3_-cube/PUCN (cyan). Three-dimensional femtosecond transient absorption spectra of (**e**) PUCN and (**f**) 5.0 wt% In_2_O_3_-cube/PUCN, (**g**) Kinetic decay curves at 670 nm for PUCN (cyan) and 5.0 wt% In_2_O_3_-cube/PUCN (red). (Reproduced with permission [51], Copyright 2024, WILEY-VCH).

Insufficient external driving force can lead to ultra-fast deep charge trapping and degradation of photocatalytic active species in carbon nitride (g-C_3_N_4_). Based on this, the David Lee et al. constructed a near-field assisted model composed of a cubic In_2_O_3_/g-C_3_N_4_ heterojunction, which is related to ultra-fast photodynamic coupling [51]. This In_2_O_3_ cube-induced near-field auxiliary system provides a catalytic “hot spot”, effectively extending the lifetime of the excited state and the shallow trapping center concentration of g-C_3_N_4_, which is conducive to increasing the concentration of active species (Figure 4a). Optical simulations combined with transient absorption spectra show that there is an intrinsic charge transfer process and longer active species lifetimes in the In_2_O_3_/g-C_3_N_4_ composite (Figure 4d–f). Photocatalytic results show that the hydrogen evolution performance driven by In_2_O_3_/g-C_3_N_4_ visible light has been improved by 34 times (Figure 4b,c). Dynamic analysis of the fs/ns-TAS results clearly shows that there is a longer shallow electron trapping process and a shorter deep electron trapping process in the In_2_O_3_-cube/PUCN heterojunction system than in the original PUCN (Figure 4g). Long-term shallow trapping and slow recombination decay allow more active photogenerated electrons to have high mobility and driving force for photocatalytic hydrogen evolution.

In many hydrogen production methods, the use of semiconductor photocatalytic water to produce hydrogen is convenient and low-cost [55,56]. In addition to g-C_3_N_4_ and TiO_2_, CdS has been proven to be one of the excellent hydrogen-producing semiconductor photocatalysts due to its narrow band gap, suitable energy band structure and good charge transfer ability [57,58,59]. One of the important challenges of using CdS in photocatalytic H_2_ production is that it is easy to be photoetched. In order to restrain this phenomenon and enhance its photostability, various strategies have been adopted to modify CdS, including loading precious metals, modifying co-catalysts, building heterostructures and surface regulation [60,61]. These strategies aim to enhance the photoresponse and accelerate the separation of photogenerated carriers, so as to improve the photocatalytic activity and prolong the photocatalytic cycle life of CdS.

**Figure 5:**
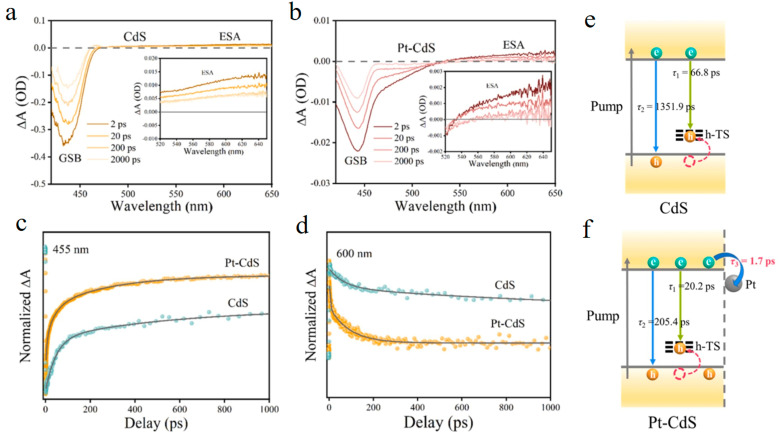
Transient absorption spectra of CdS (**a**) and Pt-CdS (**b**) under excitation at 400 nm. Kinetic decay curves of CdS (cyan) and Pt-CdS (orange) at 455 nm (**c**) and 600 nm (**d**). Schematic diagram of electron transfer between CdS (**e**) and Pt-CdS (**f**). (Reproduced with permission [63], Copyright 2024, Elsevier).

In view of the fact that the high charge recombination rate is the key factor leading to the low hydrogen production efficiency of photocatalysts. It is revealed that the charge transfer rate is of great significance to improve the hydrogen production activity of CdS-based composite photocatalysts [62]. Yu et al. studied the performance of photocatalytic hydrogen production by loading different precious metal nanoparticles on the surface of CdS hollow spheres [60]. The charge transfer rate and efficiency between them were revealed by fs-TA, which is used as a model for further investigation. CdS hollow spheres, which plays a key role in reducing the distance of electron transfer. After loading Pt on its surface, the morphology of CdS is basically unchanged. Under the excitation of 400 nm, Pt-CdS and CdS show similar spectral characteristics, with excited state absorption (ESA) and ground state bleaching (GSB) peaks of CdS appearing at 470 nm and 505 nm respectively. By fitting the dynamics at 505 nm, it was found that Pt-CdS has a higher K_e_ compared to CdS, which is attributed to the consumption of electrons on Pt accelerating the transfer of electrons. The electron flow direction was further determined by introducing an electron consumer into the system. The kinetic analysis results are consistent with the photocatalytic performance. On this basis, they used atomically dispersed Pt-CdS quantum dots prepared by a simple one-step in-situ deposition method as a model. By analyzing the kinetics of CdS and Pt-CdS, it can be seen that the electron transfer time from CdS to Pt is 1.7 ps (Figure 5a–f). The ultra-fast transfer time indicates that platinum effectively promotes the electron extraction of cadmium and simultaneously promotes photocatalytic hydrogen production and selective oxidation of 2-Thiene methanol (TM). These studies explain the charge transfer process between CdS and precious metals from a kinetic perspective, and provide theoretical guidance for the future design of CdS-based composite catalysts.

Another investigation shows that the extraction rate of holes is the key to affect the efficiency of photocatalysis, which will directly affect the conversion efficiency of solar energy to fuel. In the Yuan’s group. a ternary barbell-like CdS/MoS_2_/Cu_2_S heterojunction was prepared, with MoS_2_ nanosheets and Cu_2_S nanoparticles at both ends [64]. The hole transfer efficiency of heterojunctions was explained by fs-TA to reveal the cause of excellent photocatalytic activity (Hydrogen yield of 131.1 µmol/h). The growth of CdS terminal MoS_2_ significantly shortens the lifetime, which is the result of rapid interfacial electron transfer from CdS to the MoS_2_ reduction site. Interestingly, the loading of Cu_2_S on the other end has a more obvious impact on the charge separation and recombination process. In the CdS/MoS_2_/Cu_2_S composite, the percentage of ultrafast hole transport channels is greatly increased, further shortening the recovery life of all channels. The rapid decline in lifetime is due to the synergistic extraction of holes and electrons from CdS to Cu_2_S and MoS_2_, respectively, which reduces charge recombination. The ultra-fast charge transfer in CdS/MoS_2_/Cu_2_S is due to the existence of ultra-long charge separation state, which makes MoS_2_ accumulate more electrons and provides more opportunities for photogenerated electrons to participate in proton reduction reaction, thus improving the performance of photocatalytic hydrogen production.

**Figure 6:**
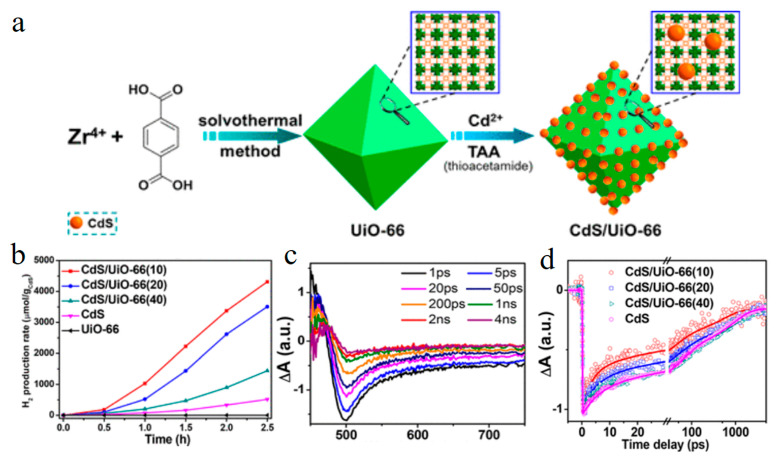
(**a**) Schematic diagram of the synthesis process of the CdS/UiO-66 composite. (**b**) Photocatalytic hydrogen production performance of different catalysts. (**c**) Transient absorption spectra of CdS/UiO-66(10). (**d**) Kinetic decay curves of CdS and composite catalyst. (Reproduced with permission [68], Copyright 2018, American Chemical Society).

Although many strategies have been taken to improve the catalytic performance of CdS, such as changing the morphology and compounding with precious metals or semiconductors, it is still a challenge to form effective CdS composite potocatalysts [61,65,66]. MOF has large specific surface area and porous structure, but its light response is relatively poor in the visible region [67]. So whether the composite of MOF and CdS can overcome their respective shortcomings and obtain catalysts with better performance? Jiang et al. used UiO-66 MOF to load CdS nanoparticles on the surface, allowing the composite to expose more adsorption centers and active sites [68]. The results of hydrogen production experiments also confirmed this (Figure 6a,b). The transient absorption results showed that the characteristic absorption and bleaching peaks of CdS appeared in the CdS/UiO-66 (Figure 6c,d). Comparative kinetics process found that CdS/UiO-66 composites had faster electron transfer and low charge recombination rates. This work provides a clearer understanding of the charge transfer between semiconductors and MOF.

In addition to forming the above types of CdS heterojunctions, it is believed that S-scheme heterojunctions can simultaneously couple photoreduction reactions and organic synthesis reactions to achieve efficient conversion [69,70,71]. However, an important part missing from our understanding is the transient dynamics of the exciton relaxation process in the S-scheme [72]. Yu et al. constructed an organic-inorganic hybrid S-heterojunction formed by loading CdS on the polymer pyrene-alt-difluorinated benzothiadiazole (PDB) [29]. Under the excitation of 400 nm, the GSB signal of CdS appeared at 510 nm, while at an earlier time, the CPDB-CdS composite also showed a negative peak at 710 nm. This peak is attributed to the charge transfer between CdS and CPDB. Combining the fluorescence and femtosecond dynamics fitting results, the charge transfer path and time between the two are known. In this work, the S-heterojunction formed by CdS and CPDB provides a platform for photocatalytic hydrogen generation and coupling with 1-phenyl-1,2-ethanediol oxidation.

Bismuth vanadate (BiVO_4_) has attracted widespread attention in the field of photocatalytic water splitting due to its cheap and readily available preparation conditions, outstanding photoresponse ability and excellent stability [70,73,74,75]. Aiming at the key scientific issue of difficulty in separating photogenerated charges in a single photocatalyst system, Li et al. developed a controllable synthesis method for BiVO_4_ square nanocrystals based on previous research, and prepared BiVO_4_ square nanocrystals exposed in different proportions of crystal faces [76]. The formation and crystal face control process of BiVO_4_ nanocrystals were studied in detail. Transient absorption spectra show that the carrier lifetime first increases and then decreases with the decrease of the thickness of BiVO_4_ nanocrystals, among which BiVO_4_-100 has the longest carrier lifetime. Therefore, photo-generated electrons and holes can be separated between different crystal faces, and the charge separation efficiency depends on the exposure ratio of different crystal faces of the catalyst.

**Figure 7:**
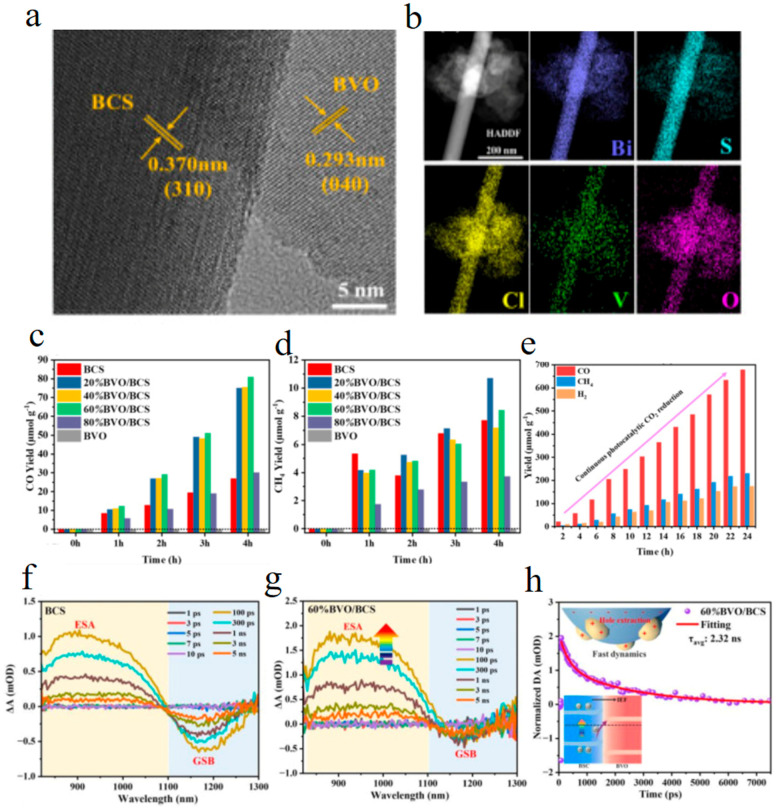
(**a**,**b**) TEM image and elemental mapping of BiVO_4_/Bi_19_Cl_3_S_27_ heterojunction. (**c**–**e**) CO and CH_4_ yields and cycle stability of BiVO_4_/Bi_19_Cl_3_S_27_. Transient absorption spectra of Bi_19_Cl_3_S_27_ (**f**) and 60% BiVO_4_/Bi_19_Cl_3_S_27_ (**g**), (**h**) decay curve of composite catalysts at 900 nm (Reproduced with permission [77], Copyright 2024, Elsevier).

Further, Li et al. designed a chemically bonded BiVO_4_/Bi_19_Cl_3_S_27_ (BVO/BCS)-S-type heterojunction with a strong built-in electric field [77]. Under light irradiation, Bi-S and Bi-O bonds are formed between Bi atoms of BVO and S atoms of BCS or Bi atoms of BVO, breaking the interface barrier and surface charge localization of Bi_19_Cl_3_S_27_ (Figure 7a,b), demonstrating high CO_2_ reduction activity (Figure 7c–e). Femtosecond transient absorption results confirm that BiVO_4_ acts as a hole trapping medium, accelerating the separation of photogenerated carriers and improving the reduction kinetics of the Bi_19_Cl_3_S_27_ surface (Figure 7f,g). This work provides a new perspective for rational design of heterojunctions to achieve efficient photoreduction of CO_2_.

Although a lot of work has been done on the research of photocatalytic reactions the separation and migration process of photogenerated charges in photocatalytic reactions spans a huge spatiotemporal scale from femtoseconds to seconds, and from atoms to microns, interpreting the process remains challenging [78,79,80,81]. Recently, Li et al. integrated a variety of full-time technologies to monitor the photogenerated charge transfer of photocatalyst nanoparticles, revealing their transfer mechanisms, and for the first time “captured” full-time images of the photogenerated charge transfer evolution [8]. By adjusting the surface ratio of Cu_2_O particles, their morphology changed from cubic to octahedral. Spatiotemporal resolved surface photovoltage measurements were performed to plot the overall charge transfer process on a femtosecond to second time scale at the level of a single particle. It was also found that photogenerated electrons were quasi-ballisticically transferred to the catalytic surface through inter-plane hot electron transfer on the sub-picosecond time scale, while photogenerated holes were transferred to the spatially separated surface and stabilized by selective capture on the microsecond time scale. This work clarifies the essential connection between the charge separation mechanism and the efficiency of photocatalytic splitting of water, and provides new understanding and research strategies for breaking through the “bottleneck” of solar photocatalytic reactions.

### 3.2. Ultrafast Kinetics of MOF in Photocatalysis

As a kind of porous crystalline materials, metal-organic framework (MOFs) has been widely studied in photocatalysis because of its clear structure, adjustable, high porosity, large surface area and so on [82,83,84]. As a new photocatalyst, MOFs can expand the photosensitive range from ultraviolet region to visible light, even to infrared light by changing its organic ligands and metal nodes [85,86,87]. In addition, the HOMO and LUMO positions of MOFs can be regulated to change its redox ability and reaction selectivity. Compared with other photocatalysts, MOFs has obvious advantages in porous structure and large surface area, and can expose more active sites in photocatalysis [88,89,90,91]. The clear structural characteristics of MOFs make it an ideal carrier to understand the structure-activity relationship. The existence of defects, metals or multinuclear clusters in MOFs composites will bring additional electron transport channels, thus accelerating the separation and transfer of photogenerated charges [92,93].

**Figure 8:**
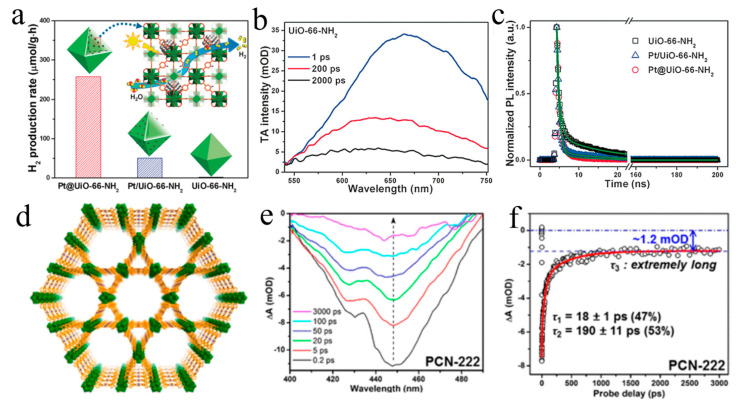
(**a**) Comparative diagram of hydrogen production performance of Pt@UiO-66-NH_2_ and Pt/UiO-66-NH_2_ catalysts. (**b**) The TA spectrum of UiO-66-NH_2_ excited by 400 nm. (**c**) Fluorescence decay curve of UiO-66-NH_2_, Pt@UiO-66-NH_2_ and Pt/UiO-66-NH_2_ catalysts (**d**) 3D network diagram of PCN-222. (**e**) TA spectra of PCN-222 excited by 500 nm. (**f**) The dynamic decay curve of PCN-222 at 515 nm (Reproduced with permission [95]. Copyright 2015, American Chemical Society).

Jiang et al. regulated the loading mode of platinum nanoparticles and UiO-66-NH_2_ MOF showing different hydrogen production performance [94]. The reason given by the ultrafast transient absorption spectra is that the Pt embedded in the MOF can shorten the electron transfer distance, which is more conducive to the separation of electron holes, thus improving the catalytic efficiency of Pt@UiO-66-NH_2_. This study not only has a better understanding of the electron transfer mechanism of metal NPs-MOF composites, but also provides a unique insight for the development of efficient MOF-based photocatalysts (Figure 8a–c). They also confirmed that porphyrin MOF has good photocatalytic activity [95]. The three-dimensional PCN-222 MOF, which is formed by connecting Zr_6_ clusters with H_2_TCPP ligands, shows excellent performance in photocatalytic CO_2_ reduction (Figure 8d). Transient absorption results show that PCN-222 can effectively accumulate long-lived photogenerated electrons, making the electron transfer of deep trap states very slow. That is to say, the existence of deep electron trap states in PCN-222 effectively suppresses electron-hole recombination. (Figure 8e,f). Compared with the corresponding porphyrin ligands, MOF significantly enhanced the photocatalytic conversion of CO_2_ to formate anions. This work provides a deeper understanding of the electron transfer mechanism involved in MOFs.

Porphyrin derivatives have become potential photosensitizers because of their important role in photosynthesis and strong absorption in the visible region [85,96,97,98,99]. The typical porphyrin unit is considered to be an important active site in photoredox reaction [96,100]. Deng et al. gave the relationship between the performance of porphyrin MOFs photocatalyst and the action site, and established a reasonable formula to quantitatively describe the distance change of the active site [101]. Taking the representative porphyrins MOF-525, PCN-221, PCN-222, PCN-223, PCN-224 and Al-PMOF as examples, the distance of their interaction sites varies obviously with the difference of cluster structure. If the active sites of porphyrin are too close to each other, the pore size of MOFs will become smaller, which will affect the kinetics of catalytic reaction. This provides more reference for the design of high efficiency photocatalyst with appropriate active site distance. It is well known that in porphyrin molecules, in addition to S_1_ fluorescence, there is also emission from the second excited singlet S_2_. The fluorescence lifetime of S_1_ is related to the polarity of solvents, and the lifetime in different polar solvents is about 1.9 to 2.1 ns [102]. The intermolecular vibrational relaxation time of various porphyrins interacting with solvents in the excited state S_1_ is between picosecond to tens of picosecond, and the intramolecular relaxation of S_2_ state is about 60–180 fs [103,104,105]. Therefore, in-depth study of the ultrafast kinetic process of porphyrin molecules can better understand its mechanism in the catalytic reaction, and provide more effective basis and guidance for the design and optimization of the catalyst [30,105,106,107,108].

**Figure 9:**
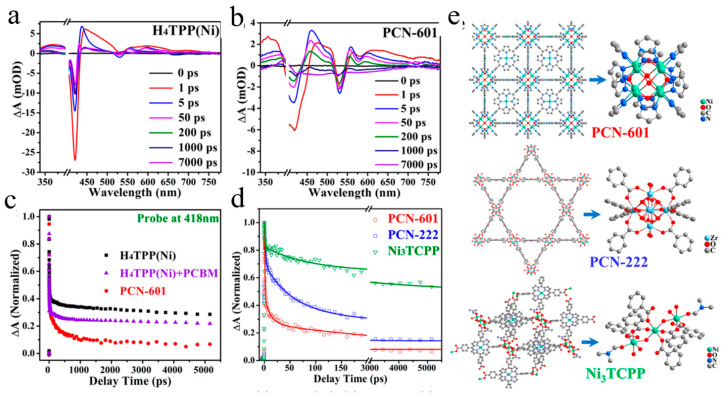
The transient absorption spectra of H_4_TPP (Ni) (**a**) and PCN-601 (**b**) under excitation at 400 nm. (**c**) The kinetic decay curves at 418 nm of H_4_TPP(Ni) (black), H_4_TPP(Ni) + PCBM (purple) and PCN-601 (red) (**d**) and PCN-601 (red), PCN-222 (blue) and Ni_3_TCPP (green), (**e**) Crystal structure and cluster structure of PCN-601, PCN-222 and Ni_3_TCPP. (Reproduced with permission [110], Copyright 2020, American Chemical Society).

In addition to hydrogen production, MOF can also be used for CO_2_ photoreduction [109]. Some classical MOFs can achieve full photocatalytic CO_2_ reduction in gas-solid phase, but the reduction efficiency is still low, which is limited by the serious recombination of electron-hole pairs of photocatalysts. This problem can be solved by reasonably designing the MOFs structure and optimizing the charge transfer dynamics. Cao et al. systematically studied the effect of strong coordination interaction between organic ligands and metal nodes on the photocatalytic reduction of PCN-601 under gas-solid conditions [110]. PCN-601 is composed of Ni-oxo clusters coordinated with pyrazole porphyrins and has stronger binding strength than PCN-222 formed by carboxylic acid porphyrin ligands (Figure 9e). When PCN-601 is used as a catalyst, the total reaction of photocatalytic reduction of CO_2_ can be realized. The products are CH_4_ and H_2_O_2_, and their activities are much better than those of PCN-222, Ni_3_TCPP and Pt/CdS. The transient absorption results show that there is no ligand-to-node charge transfer in H_4_TPP (Ni) (Figure 9a), while PCN-601 shows similar TA spectral characteristics (Figure 9b). The electron transfer rates of PCN-601, PCN-222 and Ni_3_TCPP were compared, and the GSB recovery of PCN-601 was the fastest, indicating the most effective ligand-to-node electron transfer (Figure 9c,d). Therefore, the pyrazole-NiO_x_ coordination environment has certain advantages over the carboxyl-ZrO_x_ and carboxyl-NiO_x_ configurations. These results provide insights into the relationship between the structure and properties of MOF photocatalysis.

Porphyrin MOF derivatives can also be used in the study of water splitting [111,112]. Wang et al. reported that two kinds of MOF for hydrogen production (HER-MOF) and water oxidation (WOR) were assembled into a liposome to obtain a complete hydrolysis catalyst [113]. Hydrogen-producing MOF is a kind of Hf-based MOF, in which zinc porphyrin is used as photosensitizer and Pt-porphyrin is the water reduction site. The hydrophilic WOR-MOF was constructed by using Zr-oxo cluster and BPYDC as ligands, and [Ru] and [Ir] molecular catalysts as photosensitizers and catalytic sites, respectively. Two kinds of MOFs are embedded in the hydrophobic bilayer and aqueous phase of a liposome respectively. The carrier transport between the two MOFs is connected by Fe^3+^/Fe^2+^ and TCBQ/TCBQH redox pairs to avoid charge recombination. Therefore, the whole hydrolysis process is that Fe^3+^ is used to oxidize [Ru]^2+^ in WOR-MOF, hole transfer to Ir^III^ species to produce Ir^IV/V^, and then oxidize water to produce O_2_ and release a proton. The resulting Fe^2+^ adsorbed at the lipid/water interface reacted with TCBQ to form reduced TCBQH and Fe^3+^. In HER-MOF, [(TCPP) Zn]/[(TCPP) Pt] reaches the exciton triplet state and undergoes charge separation to form [(TCPP) Zn]^+^ and [(TCPP) Pt]^−^ intermediates, [(TCPP) Zn]^+^ can oxidize TCBQH to TCBQ and release protons, and [(TCPP) Pt]^−^ protonation to [(TCPP) Pt-H], which accepts an electron and proton to produce hydrogen, thus completing the whole water splitting cycle. The apparent quantum yield is (1.5 ± 1)%. In this work, the transformation between species in the process of total hydrolysis is analyzed from the point of view of kinetics, which provides an idea for the design of total hydrolysis catalyst.

NH_2_-MIL-125 (Ti) has an important application prospect in the field of photocatalysis because of its large specific surface area and porosity, uniform pore distribution and functional controllability [114]. MIL-125 (Ti) and NH_2_-MIL-125 (Ti) MOF are isomorphic, which means that their structure and crystal properties are not affected by ligands. Although the two are isomorphic, they have some different properties, such as surface area and light absorption. During the photocatalytic process, the difference between the two systems is that NH_2_ functionalized MOFs can absorb photons with lower energy and can be used in visible photocatalysis. The charge transfer process of MIL=125 (Ti) and NH_2_-MI=125 (Ti) were studied by ultrafast spectroscopy in YAMA group [115]. The transient absorption signal of MIL-125 (Ti) is observed in the range of 500–750 nm, and the negative signal appears when the wavelength is less than 525 nm. However, NH_2_-MIL-125 (Ti) has a wide band in the range of 500–750 nm. It is obvious that the decay of excited state absorption of NH_2_-MIL-125 (Ti) is much slower than that of MIL-125 (Ti). In fact, the signal strength of more than 70% of NH_2_-MIL-125 (Ti) still exists after 3 ns, while MIL-125 (Ti) retains only 10% in the time window. The results show that -NH_2_ can not only promote the light response, but also stabilize the hole and prolong the charge lifetime.

**Figure 10:**
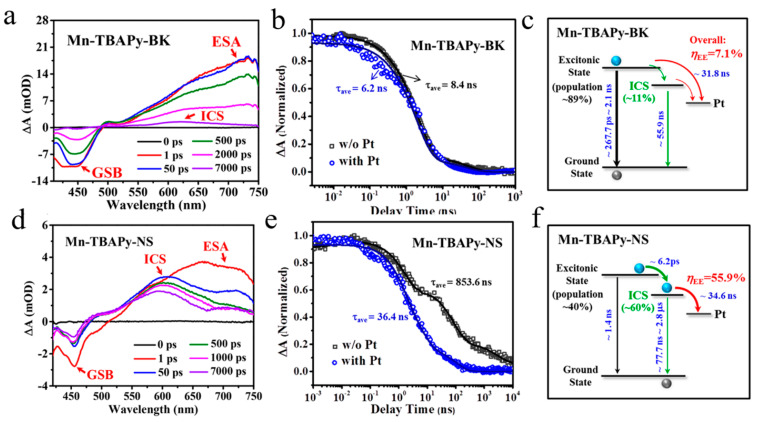
Transient absorption spectra of bulk Mn-TBAPy-BK MOF (**a**) and Mn-TBAPy-NS MOF (**d**) excited by 400 nm. The kinetic traces of Mn-TBAPy-BK (**b**) and Mn-TBAPy-NS (**e**) before and after loading Pt for 603 nm. Schematic diagram of electron transfer after Mn-TBAPy-BK (**c**) and Mn-TBAPy-NS MOF (**f**) loaded with Pt. (Reproduced with permission [119], Copyright 2022, American Chemical Society).

In recent years, the photocatalytic properties of two-dimensional metal-organic frameworks (2D MOFs) materials have been paid more and more attention [116,117,118]. However, there are few studies on the photocatalytic mechanism of 2D MOF, especially the kinetics of charge separation and extraction. Based on this, Jin et al. designed and synthesized bulk and flake Mn-TBAPy MOF for photocatalytic hydrogen production [119]. The dynamic process of carriers was revealed by TA. The block Mn-TBAPy MOF was synthesized by connecting ligand and secondary unit, and then peeled off to get flake. Under excitation at 400 nm, the bulk MOF exhibits a wide excited state absorption at 730 nm (Figure 10a). With the extension of time, the excited state absorption peak shifts from 730 nm to 630 nm. The change in this signal implies the formation of a charge-separated state (Figure 10a,d). Compared with the bulk, the charge separation state of flake MOF appears in a shorter time, and its existence time is longer. After Pt was deposited on the surface of MOF, the kinetic process of charge transfer between them was investigated (Figure 10b,e). The TA results showed that the positions of excited state absorption and charge separation states in the composite are similar to those of MOF. The electron transfer from MOF to Pt occurs in the composite, which transfers from charge separation state to Pt, and the decay of charge separation state of flake MOF is faster (from hundreds of nanoseconds to several nanoseconds) (Figure 10c,f). It has a higher efficiency of electronic extraction, which is 8 times higher than that of bulk MOF. In a word, the formation of internal charge separation state is considered to be one of the key strategies for the design and preparation of efficient photocatalyst.

Transient absorption explains the above kinetic process of MOF in photocatalytic reactions, allowing a clearer understanding of the relationship between kinetics and performance. Beyond that, UiO-66 based MOFs are commonly used in photocatalytic solar fuel production [120,121,122]. Due to the tunability of MOFs structure, it is considered to be a carrier that integrates photosensitizer and catalyst, but its performance is still limited by poor visible light absorption and short excited state lifetime of photosensitizer [123]. Therefore, Zhang et al. proposed a new strategy to significantly improve the sensitization ability of UiO-MOFs (UiO67-Irppy) [124]. By designing Ir coordination centers at the molecular level to combine with coumarin 6 (Cou 6) to form UiO67-Ir-Cou 6, their excited states are transformed from ^3^MLCT to ^3^IL states. Furthermore, Co^2+^ units were introduced into UiO67-Ir-ppy and UiO67-Ir-Cou 6 to form UiO67-Ir-ppy/M and UiO67-Ir-Cou 6/M composite photocatalysts (M = Co and Cu) to realize directional electron transfer. UiO67-Ir-Cou 6/Cu can effectively catalyze the photoreduction of CO_2_ to HCOOH with a yield of 480.7 μmol/g. The fluorescence quenching experiment confirmed the charge transfer between Ir-Cou 6 and BIH, while the triplet state of UiO67-Ir-Cou 6 was almost the same as that of UiO67-Ir-Cou 6/Co and UiO67-Ir-Cou 6/Cu. The ineffective charge transfer process from Ir-Cou 6 excited in MOFs to metal catalytic site was confirmed. The kinetic process was analyzed by nanosecond transient absorption spectroscopy. Compared with IrCou 6, after the addition of BIH, the positive absorption band of more than 500 nm disappeared and a new positive absorption peak appeared at 450 nm, indicating the formation of a new intermediate, that is, the formation of reduced Ir-Cou 6. Its decay lifetime is 186.2 μs, which is obviously longer than that of Ir-Cou 6 (82.5 ns). With the increase of Co-bpy (or Cu-bpy) concentration, the signal attenuation rate of reduced Ir-Cou 6 increases and the reduced lifetime decreases. These characteristics indicate that the interfacial electron transfer can further promote the charge transfer process in MOFs, thus improving the catalytic performance.

Immediately afterwards, Zhang et al. used multiple chromophores with different absorption bands to jointly sensitize the Cu(I) coordination center to prepare the first copper-based photosensitizer (Cu-3) with wide band and strong visible light absorption [125]. Steady and transient spectroscopy show that Cu-3 achieves a significant extension of the excited state life and a significant improvement in solar energy utilization through a double “ping-pong” energy transfer mechanism. Cu-3 was then used for energy transfer and electron transfer reactions, both of which showed excellent catalytic activity. This work provides important scientific reference for the construction of efficient non-noble metal photosensitizers with broad bands, strong visible absorption and long excited state lifetimes.

### 3.3. Ultrafast Kinetics of Halide Perovskite in Photocatalysis

**Figure 11:**
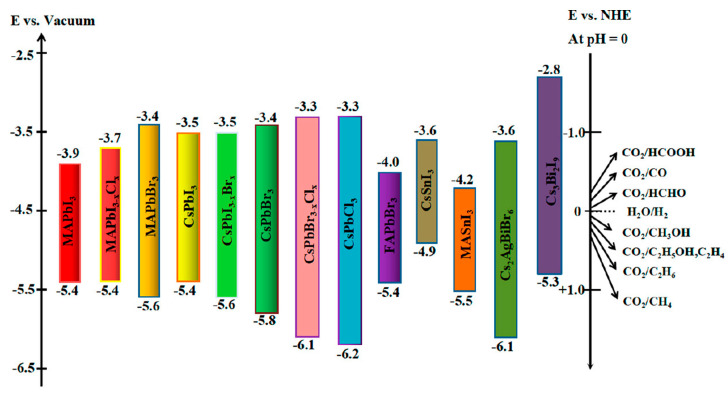
Band edge position of different halide perovskite relative to normal hydrogen electrode (NHE) (Reproduced with permission [138] Copyright 2021, American Chemical Society).

Many semiconductors can be used as photocatalysts for CO_2_RR, including metal oxides (TiO_2_, Cu_2_O, etc.), sulfides (CdS, CuIn_5_S_8_, etc.), nitrides (CoN, g-C_3_N_4_, etc.), metal-organic framework (MOFs), perovskite oxides and so on [126,127,128,129]. The redox potential of some commonly used semiconductor photocatalytic CO_2_ reduction to C1 products is shown in Figure 11. Although these materials have been used in CO_2_RR, they still face some problems, such as poor stability, limited photoresponse, weak reaction activity, and most materials can only absorb ultraviolet rays and have low selectivity for the products [130,131,132,133]. Therefore, in order to solve these problems, it is necessary to develop new photocatalysts with wide range of light response. From the point of view of thermodynamics, the appropriate band structure of the catalyst plays an important role in the redox potential of the reaction. The CB of perovskite quantum dots (QDs) is more negative than that of traditional semiconductors, which means that perovskite is more beneficial to the reduction of CO_2_ [131,134,135]. However, almost all perovskites (PVKs) quantum dots are difficult to react at the oxidation end. Most traditional semiconductors have a certain potential for oxygen production, so they are suitable candidates for forming heterojunctions with halide perovskite quantum dots [136,137,138]. PVK can be used for photocatalytic CO_2_ reduction to produce all C1 products [139]. In fact, the photocatalytic reduction of CO_2_ is affected by thermodynamic and kinetic factors. For example, although the process of reduction of CO_2_ to CO or HCOOH only involves 2H bonding to 2e^−^, the bond formation from CO_2_ to CO or HCOOH is limited by kinetics. It is more complicated to realize the conversion of CO_2_ to CH_3_OH, which involves more coupling of electrons and protons.

Zero-dimensional (0D) PVK quantum dots, also known as semiconductor nanocrystals, have the characteristics of large specific surface area, high atomic utilization, quantum confinement effect and so on [140]. In the application of photocatalysis, quantum dots show obvious advantages, such as high extinction coefficient, adjustable band gap and short charge transfer path [141]. Because of their unique properties, quantum dots are expected to be candidates for CO_2_ reduction [137,142]. However, the high photoluminescence quantum yield of PVK QDs and its instability to light, heat, oxygen and water limit the application in the field of photocatalytic CO_2_ reduction [143,144,145]. Therefore, various heterojunction composites are constructed to enhance the photogenerated carrier separation and photocatalyst stability to achieve efficient CO_2_ reduction [53,116,146,147,148,149,150,151,152].

**Figure 12:**
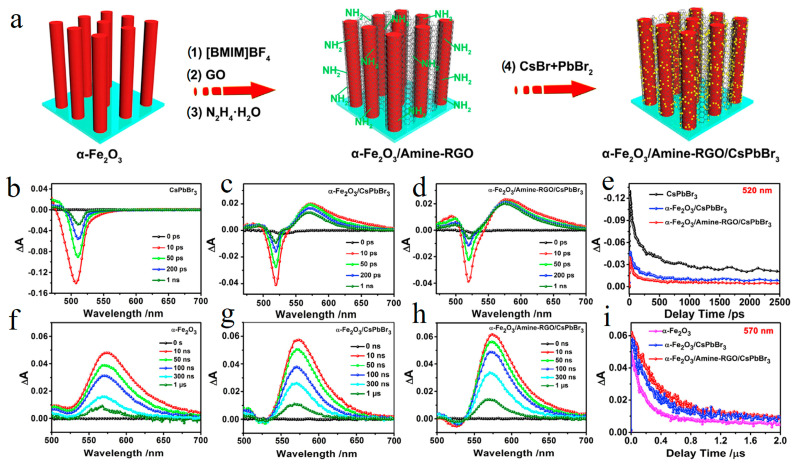
(**a**) preparation process of α-Fe_2_O_3_/Amine-RGO/CsPbBr_3,_ (**b**) CsPbBr_3_, (**c**) α-Fe_2_O_3_/CsPbBr_3_, (**d**) α-Fe_2_O_3_/Amine-RGO/CsPbBr_3_ recorded TA spectra on sub-nanosecond time scale and TA kinetic curve at 520 nm (**e**). (**f**) α-Fe_2_O_3_, (**g**) α-Fe_2_O_3_/CsPbBr_3,_ (**h**) α-Fe_2_O_3_/Amine-RGO/CsPbBr_3_ recorded TA kinetic curve on microsecond time scale. (**i**) The kinetic curve of TA was recorded at 570 nm and the excitation wavelength was 400 nm (Reproduced with permission [151], Copyright 2020, Elsevier).

Kuang et al. further designed and synthesized perovskite-based Z-scheme heterojunction α-Fe_2_O_3_/Amine-RGO/CsPbBr_3_ for photocatalytic CO_2_ reduction [151]. Compared with CsPbBr_3_, the effective separation of photogenerated carriers in Z-scheme system improved the photocatalytic performance by 8.3 times, and the product yield reached 469.16 mmol·g^−1^ (the products were CH_4_, CO and H_2_) (Figure 12a). The charge transfer kinetics of Z-scheme was explored by ultrafast spectroscopy. By comparing the kinetics of CsPbBr_3_ QDs with that of α-Fe_2_O_3_/Amine-RGO/CsPbBr_3_, it was found that the signal at 520 nm was GSB of CsPbBr_3_, while the positive signal at 570 nm was the ESA of Fe_2_O_3_ (Figure 12b–d,f–h). The kinetic decay curve of CsPbBr_3_ at 520 nm was fitted, and it was found that the composite had a faster decay process (Figure 12e). By fitting the kinetic curve of Fe_2_O_3_ at 570 nm, it is found that the hole lifetime is longer (Figure 12i). This phenomenon also supports the Z-scheme charge transfer process between CsPbBr_3_ and α-Fe_2_O_3_, so this work provides a useful inspiration for adjusting carrier dynamics to improve the photocatalytic performance of perovskite.

**Figure 13:**
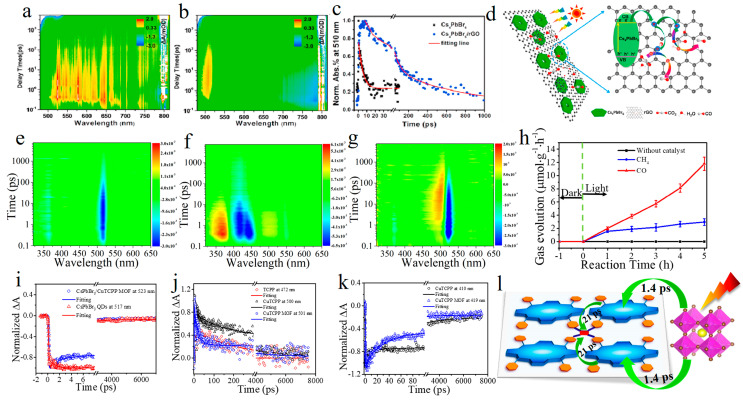
Transient absorption spectra of Cs_4_PbBr_6_ (**a**) and Cs_4_PbBr_6_/rGO (**b**), (**c**) Decay curves of sample normalized at wavelength 510 nm of Cs_4_PbBr_6_ (black) and Cs_4_PbBr_6_/rGO (blue). (**d**) CO_2_ reduction mechanism diagram of Cs_4_PbBr_6_/rGO as catalyst (Reproduced with permission [153], Copyright 2020, Elsevier). The transient absorption spectra of the CsPbBr_3_ QDs (**e**), CuTCPP MOF (**f**), CsPbBr_3_/CuTCPP MOF (**g**) excited at 300 nm. (**h**) Time-dependent yield of CO and CH_4_. Decay curves are normalized to the maximum signals of CsPbBr_3_ QDs (red) CsPbBr_3_/CuTCPP MOF (blue) at ∼517 nm (**i**), TCPP (red), CuTCPP (black) and CuTCPP MOF (blue) at ∼500 nm (**j**) CuTCPP (black) and CuTCPP MOF (blue) at ∼410 nm (**k**). (**l**) Electron transfer mechanism diagram of CO_2_ reduction using CsPbBr_3_/CuTCPP MOF as catalyst (Reproduced with permission [154], Copyright 2021, WILEY-VCH).

Zhu et al. reported that Cs_4_PbBr_6_/rGO as a catalyst for CO_2_ reduction improved the selectivity of CO because the oxygen vacancy on the surface of rGO played an important role [153]. The charge transfer process between Cs_4_PbBr_6_ and rGO is studied by fs-TA. It is found that there is a ground state bleaching peak of Cs_4_PbBr_6_ at 510 nm (Figure 13a,b). Compared with Cs_4_PbBr_6_ (5 ps), the Cs_4_PbBr_6_/rGO has two longer lifetimes (τ_2_: 64.9 ps (39.9%), and τ_3_: 752 ps (36.6%), because the appearance of the defect state on the surface of rGO prolongs the recombination of charge and hole (Figure 13c). Therefore, the photoexcited Cs_4_PbBr_6_ transfers electrons to the defect state on the surface of rGO, which improves the catalytic activity (Figure 13d). In order to reveal the charge transfer kinetics between perovskite and MOF and within MOF, Wang et al. designed cascade electron transfer catalyst to improve photocatalytic performance [154]. CsPbBr_3_ QDs and 2D CuTCPP MOF were combined in situ to form 0D/2D composite, which showed high catalytic activity (the yields of CO and CH_4_ were 11.8 and 2.95 µmol·g^−1^l·h^−1^, respectively) (Figure 13h). fs-TAS reveal that the “cascade electron transfer” stimulates the interfacial and internal electron transfer process of the catalyst (Figure 13e–j,i–k). It takes only 1.4 ps for electrons to transfer from the CsPbBr_3_ QDs to the MOFs, and then completes the electron transfer from ligands to nodes in the CuTCPP MOFs within 21 ps (Figure 13l). This ultra-fast electron transfer is the key to improve the catalytic performance. This work provides a useful insight for improving the efficiency of photocatalysis from perspective of kinetics.

**Figure 14:**
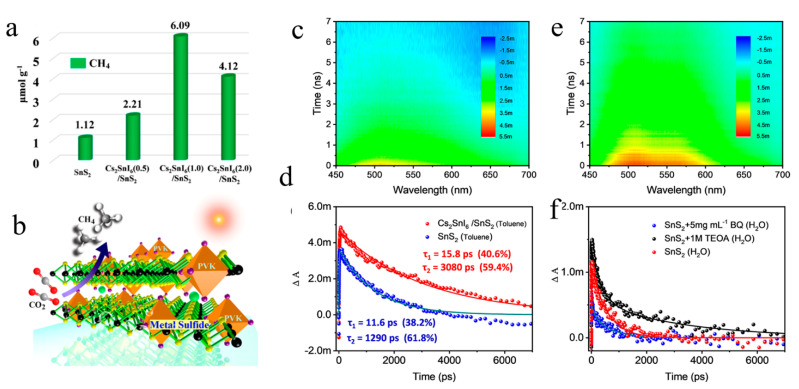
Photocatalytic performance of for CO_2_ reduction: (**a**) comparison of CH_4_ and CO yield using different catalysts; (**b**) Schematic diagram of photocatalytic CO_2_ reduction with Cs_2_SnI_6_/SnS_2_ catalyst. Contour maps of transient absorption spectra of the SnS_2_ (**c**), Cs_2_SnI_6_/SnS_2_ (**e**) excited at 300 nm. (**d**) Kinetic decay curves of SnS_2_ (blue) and Cs_2_SnI_6_/SnS_2_ (red) in toluene solution. (**f**) Kinetic curves of SnS_2_ in aqueous solution (BQ (bule), TEOA (black) and H_2_O (red)) (Reproduced with permission [155]. Copyright 2019, American Chemical Society).

The kinetics of lead-based halide perovskite has been studied, so does lead-free halide perovskite have a similar kinetic process? Kuang et al. then synthesized a new type of Sn atom-shared Cs_2_SnI_6_/SnS_2_ heterojunction in situ, which showed better performance by photocatalytic CO_2_ [155]. Combined with DFT calculation, TA and KPFM, a type II Cs_2_SnI_6_/SnS_2_ heterojunction with close contact interface is found, which can effectively promote carrier transfer (Figure 14a,b). The TA results show that there is a positive absorption peak at 525 nm in SnS_2_ and the composite catalyst, which belongs to the photogenerated electrons of SnS_2_ (Figure 14c). After the introduction of Cs_2_SnI_6_, the increase of PIA intensity and the delay of dynamic decay can be explained as the prolongation of the lifetime of photogenerated electrons in SnS_2_ (Figure 14e). Then the transfer direction of electron and hole is determined by adding sacrificial agents to the system. That is, the electron of Cs_2_SnI_6_ is transferred to SnS_2_, while the hole of SnS_2_ is transferred to Cs_2_SnI_6_ (Figure 14d,f). This explains the improvement of catalytic performance after the formation of type II heterojunction. Therefore, the in-situ construction of heterojunctions by atomic sharing strategy will broaden the synthesis of various perovskite-based materials (Cs_3_M_2_X_9_/M_2_Y_3_, M = Bi, Sb, X = Br, I and Y = S, Se) and expand their applications in the fields of electronics, solar cells, photodetectors, photocatalysis and so on.

**Figure 15:**
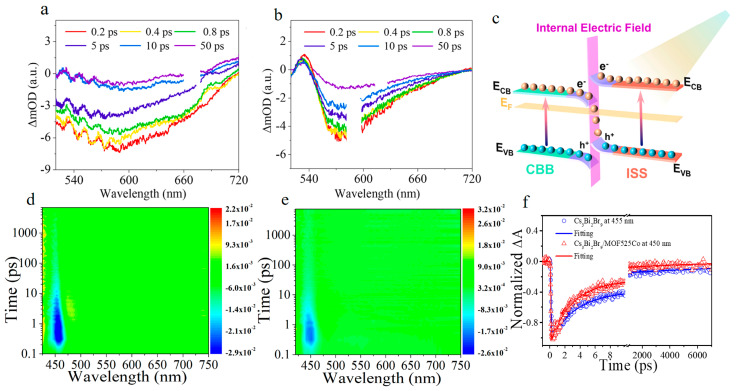
The fs-TA spectra of ISS (**a**) and CBB (**b**), (**c**) S-scheme charge transfer mechanism formed between ISS and CBB. (Reproduced with permission [156], Copyright 2022, Elsevier) Contour maps of the transient absorption spectra of Cs_3_Bi_2_Br_9_ QDs (**d**), Cs_3_Bi_2_Br_9_/MOF 525 Co (**e**) excited at 400 nm; (**f**) Decay curves were normalized to the maximum signals at wavelengths of ∼450 nm. (Reproduced with permission [157]. Copyright 2023, The Royal Society of Chemistry).

In addition to tin-based perovskite, bismuth-based perovskite has some potential for CO_2_ photoreduction. In the Fu’s group, the In_4_SnS_8_/Cs_3_Bi_2_Br_9_ heterojunction was assembled in situ by using In_4_SnS_8_ (ISS) nanoflowers and Cs_3_Bi_2_Br_9_ (CBB) quantum dots, and the composite showed excellent performance in CO_2_ reduction [156]. The yield and selectivity of CO were 9.55 μmol/g/h and 92.9%, respectively, which were 3.8 and 1.5 times higher than that of ISS. Femtosecond transient absorption spectra further investigated the direction of electron transfer between them. Under excitation at 400 nm, both of them showed a GSB peak of ISS near 580 nm. The difference is that the composite has a positive absorption peak at 540 nm, indicating that the holes on the surface of ISS recombine with the electrons of quantum dots (Figure 15a,b). In addition, the S-scheme heterojunction between ISS and CBB was confirmed by other photochemical and photophysical methods (Figure 15c). Therefore, under the condition of light, the built-in electric field formed at the interface of the ISS/CBB heterojunction promotes the hole transfer from the VB of ISS to the CB of CBB. This study confirms that the formation of built-in electric field in S-scheme heterojunctions can improve the selectivity of CO.

On this basis, in order to further regulate the selectivity of CO_2_ photoreduction, Wang et al. continued to load Cs_3_Bi_2_Br_9_ quantum dots (CBB QDs) on MOF 525 Co to form Cs_3_Bi_2_Br_9_/MOF 525 Co heterojunction, which made the selectivity of CO up to 99.5% for CO_2_ photoreduction [157]. The charge transfer kinetics between them is revealed by fs-TA. Under excitation at 400 nm, the three-dimensional transient absorption spectra of CBB QDs and Cs_3_Bi_2_Br_9_/MOF 525 Co show that there is a strong negative signal at 450 nm and a weak positive signal near 470 nm, which is classified as GSB and ESA peak of CBB, respectively (Figure 15d,e). By analyzing their dynamics at 455 nm, it is known that the charge transfer time from CBB to MOF 525 Co is 136 ps (Figure 15f). Effective charge separation and ultra-long charge recombination time are more conducive to the improvement of catalytic performance. This work reveals the charge transfer of the catalyst in the illumination process from the point of view of kinetics, which provides a reference for the subsequent design and synthesis of catalysts with high carrier separation efficiency.

## 4. Summary and Perspectives

High spatio-temporal resolution spectroscopy plays an irreplaceable role in revealing the photocatalytic process and mechanism. This review summarizes the principle of femtosecond transient absorption spectroscopy, the dynamic processes of common semiconductors, and the application of femtosecond spectroscopy in photocatalysis. On the one hand, in most photocatalytic reactions, researchers have monitored the multi-step, ultra-fast electron transfer process of the reaction at high temporal resolution by combining fluorescence lifetime, femtosecond transient absorption spectroscopy, or laser flash photolysis. In addition to the outstanding achievements previously achieved, the application of ultrafast spectroscopy faces many challenges in the field of photocatalysis. For example, few systems can detect the charge separation state of the photocatalyst, making it difficult to determine the true catalytic process conversion; On the other hand, fs-TA spectroscopy can only resolve kinetic information, and it is difficult to describe the real-time conversion process of the catalyst. In order to overcome current challenges, we propose the following future research directions and solutions:

(1) Based on the in-depth study of the micro-kinetic behavior of photo-generated charge in semiconductor catalyst and reaction system, the driving force of photo-generated charge separation, the key factors determining the initiation reaction are clarified. The relationship between the results of photo-generated charge behavior and photocatalytic performance will be established to realize the understanding of the complex mechanism of photocatalysis. It can provide new ideas and research methods for rational design of photocatalytic materials with better performance. To sum up, it is very important to understand the research methods of photo-generated charge behavior. For example, combining ultrafast spectroscopy with full-time surface photovoltage technology can provide the entire process of photo-generated charge separation and migration.

(2) Transient absorption spectra can not only obtain the kinetic information of excited states of materials, but also provide more means for fully understanding the microscopic properties of materials when combined with other techniques. For example, when combined with microscopic imaging technology, the dynamic time scale of excited state can be detected, and the diffusion and propagation behavior of excited state can also be observed. Through these information, we can further understand the influence of excited state dynamics on the performance of photocatalysts. Usually, the research object of fluorescence microscope is limited to luminescent materials, but the combination of microscope and transient absorption technology can observe the absorption of materials to study the micro-mechanism, which greatly expands the research object and research field.

(3) Build a spectral detection method with temporal and spatial resolution. Time-resolved spectroscopy can realize the analysis of the dynamics in the material, while spatial resolution can realize the detection of properties of different parts of the material. Combining time and spatial resolution can obtain the dynamic processes of different parts of the material, thereby analyzing the surface states and edge states of the material. etc. For example, using high-resolution confocal fluorescence microscopy combined with time-correlated single photon counting (TCSPC) can explore the dynamics at different positions of the luminescent material and obtain information on the surface state of the material based on the different dynamics. Transient absorption microscopy provides a means to track the evolution of optically excited states in time and space. It can not only spatially overlap pump pulses and probe pulses to obtain information similar to the overall transient absorption test, but also fix a specific delay time, keep pump pulses and probe pulses spatially overlap and scan the sample, providing spatial changes in the excited state dynamics of the entire sample; It is also possible to place the pump and probe pulses in different positions to explore the information about the evolution in space of the optically excited states generated at specific positions.

In summary, femtosecond transient absorption spectroscopy can not only provide rich information on photophysical and photochemical processes for studying single-component systems, and build a population evolution picture of particles with different energy states after light excitation, but also be used to clarify the charge transfer and energy transfer processes between substances. The ultrafast photonics related processes have important applications in the fields of photocatalysis, solar cells, LEDs, nonlinear optics, photodetectors, etc. and can provide theoretical support and experimental verification for the screening of device materials selection and structural design.

## Data Availability

No new data were created or analyzed in this study. Data sharing is not applicable to this article.
